# How Spatial Variation in Areal Extent and Configuration of Labile Vegetation States Affect the Riparian Bird Community in Arctic Tundra

**DOI:** 10.1371/journal.pone.0063312

**Published:** 2013-05-14

**Authors:** John-André Henden, Nigel G. Yoccoz, Rolf A. Ims, Knut Langeland

**Affiliations:** 1 Department of Arctic and Marine Biology, University of Tromsø, Tromsø, Norway; 2 Department for Arctic Ecology, Norwegian Institute for Nature Research, The FRAM Centre, Tromsø, Norway; Lakehead University, Canada

## Abstract

The Arctic tundra is currently experiencing an unprecedented combination of climate change, change in grazing pressure by large herbivores and growing human activity. Thickets of tall shrubs represent a conspicuous vegetation state in northern and temperate ecosystems, where it serves important ecological functions, including habitat for wildlife. Thickets are however labile, as tall shrubs respond rapidly to both abiotic and biotic environmental drivers. Our aim was to assess how large-scale spatial variation in willow thicket areal extent, configuration and habitat structure affected bird abundance, occupancy rates and species richness so as to provide an empirical basis for predicting the outcome of environmental change for riparian tundra bird communities. Based on a 4-year count data series, obtained through a large-scale study design in low arctic tundra in northern Norway, statistical hierarchical community models were deployed to assess relations between habitat configuration and bird species occupancy and community richness. We found that species abundance, occupancy and richness were greatly affected by willow areal extent and configuration, habitat features likely to be affected by intense ungulate browsing as well as climate warming. In sum, total species richness was maximized in large and tall willow patches of small to intermediate degree of fragmentation. These community effects were mainly driven by responses in the occupancy rates of species depending on tall willows for foraging and breeding, while species favouring other vegetation states were not affected**.** In light of the predicted climate driven willow shrub encroachment in riparian tundra habitats, our study predicts that many bird species would increase in abundance, and that the bird community as a whole could become enriched. Conversely, in tundra regions where overabundance of large herbivores leads to decreased areal extent, reduced height and increased fragmentation of willow thickets, bird community richness and species-specific abundance are likely to be significantly reduced.

## Introduction

Thickets of tall shrubs constitute a conspicuous vegetation state in northern and some temperate ecosystems, where it serves important ecological functions, including habitat for wildlife [1]–[3]. Such thickets are typical for riparian areas where they form habitat mosaics together with often more short-statured, alternative vegetation states such as meadows. Such habitat mosaics composed of more productive vegetation with highly contrasting structural characteristics make riparian zones biodiversity hotspots in northern and temperate ecosystems [4].

Willow (*Salix* spp.) is often the dominant thicket-forming plant in riparian zones [4]–[6], especially in the Arctic. Even though riparian willow thickets often comprise only a small portion of the landscape, biodiversity associated with this habitat type is often higher than in adjacent habitats [3], [7], [8] or they contribute to the regional diversity by harbouring distinctly different species assemblages [9]. Riparian willow thickets are particularly important for birds [5], [10], [11], with species richness in temperate riparian habitats with willows up to 10–14 times higher than that of adjacent non-riparian habitats ([5]; and references therein). Moreover, Baril et al. [5] found that bird richness in the northern range of Yellowstone National Park was close to three times higher in tall willows than in willows vertically suppressed by ungulates. Hence, willow forming thickets represent a vegetation state of high conservation value because a potentially large portion of the regional flora and fauna can be preserved within their bounds.

The Arctic tundra is currently experiencing an unprecedented combination of climate change, changing grazing pressure by large herbivores as well as increasing human activity [12]–[16]. Willow thickets are functionally important, but labile components of many ecosystems because they have been shown to respond rapidly (i.e. over a few decades) to both abiotic and biotic environmental drivers [17], [18]. Recently, there have been many studies focusing on the dynamics of willow thickets both in Arctic [14], [19]–[21] and temperate ecosystems [3], mainly for two reasons. On one hand, an expansion in willow thicket areal extent has been documented and predicted to accelerate in parts of the Arctic, mostly attributed to a warming climate [14], [19], [22], [23]. On the other hand, pervasive effects of ungulate overabundance dramatically reduce palatable shrubs in many ecosystems [24]–[28]. Because of their high palatability to many ungulate species, several studies have shown that ungulate browsing is capable of reducing the cover and height of willows [25], [29]–[32]. Other attributes of the spatial configuration of the willow thickets may also be affected by intense browsing. Specifically, studies targeting the states of riparian vegetation in low arctic tundra in north-eastern Norway [7], indicate that high abundant ungulates are responsible for fragmentation of tall thicket complexes through a shredding effect [33], causing both loss of thicket area and more edges against the surrounding alternative meadow vegetation state. In parts of the Eurasian Arctic tundra ungulates, particularly reindeer (*Rangifer tarandus*), have increased substantially during the last 20–30 years [15], [26], [34], [35]. Accordingly, there is evidence from Fennoscandia that heavy grazing by reindeer may hamper deciduous shrub growth [32], [36], and thereby in effect keep the tundra vegetation in a short-statured state [37].

Habitat loss and fragmentation are generally taken as the greatest threats to global biodiversity and are often highlighted as the major processes leading to landscape change [38]–[40]. Both theoretical and empirical studies have often found habitat area to be the most important predictor of species occurrence and richness in fragmented landscapes, with its effect consistently positive and strong across regions, habitats and taxa [41]–[47]. However, habitat area may not sufficiently explain the effects of shredding or the breaking apart of habitat, since patches of equal area, but different configuration, may not necessarily hold the same qualities for species or animal communities. Accordingly, some studies have found that variables quantifying both the area and shape of a patch are often the strongest predictors of both species occupancy and overall species richness [42], [45]. The effects of area and configuration of habitat patches are often conflated [48] as they tend to co-occur in natural settings. Yet, decomposing the relative effects of these two processes may be important for conservation and management as they warrant different actions [48]. For instance, if a particular species or species assemblage is mainly affected by habitat area, then habitat protection or restoration would be the sensible action, whereas if species are sensitive to the breaking apart of a particular habitat, then the aim would be on altering its spatial configuration (i.e. increased importance of site-level management).

Our aim with this study was to assess how varying willow thicket areal extent and configuration in mosaics with more short-statured vegetation states (meadows or heaths) affect bird species richness and occupancy rates. Given the paucity of bird time series from the Arctic, that are long enough to encompass temporal vegetation state shifts [35], “space-for-time” approaches currently provide the only empirical means to derive predictions about such effects. How bird species or species assemblages are expected to respond to shifting vegetation states likely depends on the strength of their affiliation to a specific habitat. Indeed, both species tightly linked to tall thicket-forming shrubs and species affiliated with short-statured alternative vegetation states (like meadows) are predicted to respond, albeit with opposite sign. Furthermore, species that depend on more than one vegetation state (e.g. for foraging and nesting) may benefit from high patch heterogeneity [49]. Published knowledge of habitat-species relations for low arctic tundra bird communities is scarce and mainly found in a few qualitative natural history accounts which hamper derivation of precise predictions.

## Materials and Methods

### Study Area

The study was carried out in three regions in the north-eastern part of Finnmark, northern Norway from 2005 to 2008. Two regions were situated at the Varanger Peninsula, between 70–71° N and 28–31°E and one on Laksefjordvidda (70–71° N, 27°E) about 100 km west of Varanger peninsula [50]. At the Varanger peninsula, the selected study sites were situated in the main river valleys of Vestre Jakobselv (VJ) and Komag (KO), ∼40 km apart, whereas at Laksefjordvidda the study sites were situated along the mountain pass Ifjord (IF). According to Moen [51], the three regions are situated within an intermediate oceanic vegetation sector, with annual yearly temperatures ranging 0 to - 4°C and annual precipitation varying between 400–1000 mm. The northernmost part of the Varanger peninsula is classified as southern arctic low-shrub tundra [52]. However, the three study sites hold the same main vegetation characteristics [53]. The vegetated areas are dominated by open heaths mainly composed of dwarf shrubs such as *Empetrum nigrum*, *Betula nana, Vaccinium* spp. and lichens [26], [53], [54]. In moist depressions and especially on sediment plains along creeks and rivers, lusher meadows interspersed with patches of willow thickets (mainly *Salix lapponum, S. phylicifolia, S. lanata* and *S. glauca*) occur.

### Study Design and Bird Count Method

We strategically selected a total of 37 sampling points (12 in KO, 13 in VJ and 12 in IF) on riparian sediment plains where tall willow thickets are embedded in more short-statured, herbaceous meadow vegetation, in order to cover the existing variation in willows thicket size and configuration. In order to cover both of these vegetation states each sampling point was located at the edge between a willow thicket patch and the surrounding meadow. The bird counts were made within a radius of 100 m from each point. Because the sediment plains were quite narrow this circle also included a portion of the adjacent heaths. Thus our sampling included the three main vegetation states in riparian valleys of the study regions (willow thickets, meadows and heaths). The distance between two adjacent sampling points was on average 623 meters (SD = 530 meters, range: 164–2248 m) and there was only one case where the sampling scale of two neighbouring points overlapped.

We censused birds by point sampling method [55] during a 3–5 day period in early July in the years 2005 to 2008. Due to the short summer season at this high latitude, the timing of the census corresponds to the early part of the breeding season and thus the peak activity period for all bird species. Each sampling point was visited 3 times each year, but not on days with wind or rain. At these high latitudes the sun never sets in the period of census, and birds sing most actively in the evening from 19.00–23.00 and in the morning from 02.00–10.00 [56], [57]. Accordingly, bird counts were primarily conducted in these periods. Moreover, each observer alternated the timing of the censuses between the different sampling points such that no sampling points were consistently sampled later or earlier during the sampling period or day, than others. The recording period for each census was set to fifteen minutes, but started five minutes after arrival at the point. In this study the same two experienced observers conducted the sampling during the four years, one covering both Komag and Ifjord, while the other covered Vestre Jakobselv. To get an indication of the habitat use of the different bird species in the field, the observer noted the type of habitat in which they were first detected. For this purpose the following categories were used: willow thicket, meadow, heath, water, and “flying over” (Table 1). Furthermore, we used two qualitative natural history accounts relevant for our geographic region to classify the different bird species into three groups based on their *a priori* expected affiliation to willow thickets as habitat both for breeding and foraging [57], [58] (Table 1). The three assemblages were; 1) Willow Canopy-Breeding (WCB) species that breed and forage in the thickets, 2) Willow related Ground-Breeding (WGB) species that breed on the ground and forage in the thickets and, 3) species with a looser connection to thickets, mostly birds affiliated to the adjacent Open Tundra (OT). Note that gulls and birds associated with shorelines were removed from further analyses because of their lack of strong affiliation to willow thickets and the open tundra.

**Table 1:** The number of times the different species were detected in the 5 habitat categories during the 4 year study as well as the total number of observations for each species in the course of the study (i.e. SUM).

Latin name	Common name	Assemblage	Heath	Thickets	“Flying over”	Meadow	Water	SUM
*Fringilla montifringilla*	Brambling	WCB	0	13	0	0	0	13
*Carduelis flammea*	Common Redpoll	WCB	25	466	185	1	0	677
*Turdus pilaris*	Fieldfare	WCB	13	125	27	10	0	175
*Turdus iliacus*	Redwing	WCB	16	201	9	4	0	230
*Luscinia svecica*	Bluethroat	WGB	1	102	0	2	1	106
*Emberiza schoeniclus*	Reed Bunting	WGB	0	19	0	0	0	19
*Calidris temminckii*	Temminck’s Stint	WGB	0	5	7	18	1	31
*Lagopus lagopus*	Willow Ptarmigan	WGB	1	16	1	3	0	21
*Phylloscopus trochilus*	Willow Warbler	WGB	1	288	1	0	0	290
*Pluvialis apricaria*	Eurasian Golden Plover	OT	44	3	0	3	4	54
*Calcarius lapponicus*	Lapland Bunting	OT	56	126	2	15	0	199
*Stercorarius longicaudus*	Long-tailed Skua	OT	9	2	20	0	0	31
*Anthus pratensis*	Meadow Pipit	OT	257	213	16	76	0	562
*Oenanthe oenanthe*	Northern Wheatear	OT	18	8	0	5	0	31
*Anthus cervinus*	Red-throated Pipit	OT	6	23	2	2	0	33
*Buteo lagopus*	Rough-legged Buzzard	OT	9	0	1	0	0	10
*Motacilla alba*	White Wagtail	OT	16	23	8	5	8	60

Each bird was allocated to three assemblages based in a priori expectations regarding their habitat preferences. WCB denote Willow Canopy-Breeding, WGB denote Willow Ground-breeding and OT denote Open Tundra species.

### Quantifying Willow Thicket Area and Fragmentation

We derived willow thicket area and fragmentation variables from 1∶15000 ortho-rectified aerial photographs (in raster tiff-format), taken in the summer of 2006. The pixel resolution of the photographs was 0.20 meter. For converting aerial photographs from tiff-format to img-format, ARC GIS-software, version 9.1 [59] was used. All willow thickets within the different study areas were digitized in GRASS, version 6.1 [60] and appropriate raw data files were assembled by the same software. The raw data were further analysed using FRAGSTATS, version 3.3 [61]. Area-based willow thicket characteristics were quantified within a 200×200 meter (4 ha) quadrate centred on the sampling point at the edge of the thicket, corresponding to the bird point count detection radius of 100 meters. To quantify area-based willow thicket characteristics we extracted willow thicket extent, and two variables describing the degree of fragmentation that are straightforward to interpret in terms of biological significance for birds. The two fragmentation variables were *patch density* (Pd) and *edge density* (Ed), for which increasing values indicate increasing fragmentation. The willow thicket extent variable was taken as the percentage of the area (4 ha) covered by willows, hereafter referred to as *area*. For all analyses, we defined a willow patch as consisting of an aggregation of pixels that are spatially connected using the eight neighbours rule [61]. Patch density and edge density were then quantified as the number of distinct patches (>2 m apart) and the number of meters of edge, respectively, within the measurement scale.

Additionally, we measured two local variables which define the local habitat structure, which were willow thicket height (Wheight) and density (Wpf). The two variables were measured at four points along a 15 m interval (including the sampling point) on the border between the thicket and the surrounding meadow at each sampling point, assumed to be representative for the scale of the area-based measures. Thicket density was assessed by a modified point frequency method, placing a telescope stick vertically 1 m inside the thicket and counting the number of hits with secondary stems and branches. Thicket height was measured as the highest willow branch inside a circle with 20 cm radius surrounding the telescope stick. The sampling point score for the height and density variables was the mean of the four measurements. The range of the willow thicket variables were highly overlapping between the study regions (Table S2 in Appendix S1). Accordingly, there were no consistent differences between the regions in the areal extent and degree of fragmentation of the willow thickets that could confound these variables with regional differences within the bird community. Exploratory analyses of the willow thicket configuration variables showed that the fragmentation variables patch density and edge density were strongly correlated (Pearson correlation >0.7), whereas the others were not or only slightly correlated (Pearson correlations <0.43).

### Data Analyses

For all the analyses we reduced counts of individual birds to simple “detection/nondetection” (1/0) for each visit to a sampling point in each year of the study. Further, because of the high correlation (>0.7) between patch density and edge density, we opted to use only edge density in the analysis because this variable better represents the shape of the habitat and the effect of reindeer browsing (shredding effect cf. [7]). For the analysis of species-specific occupancy and community richness, we adopted the multispecies-multiyear hierarchical model presented in Kéry et al. [62]. One of the strengths of using such hierarchical models is that various biological and observation/sampling components can be specifically formulated and related to one another [62], [63]. For instance, when estimating species occupancy and subsequently richness, such models aid in distinguishing true absence from non-detection. This is formally done by specifically incorporating presence-absence and detection-nondetection (depending on whether a species is actually present) as two distinct components in the model [45], [62], [64]. We followed the approach of Kéry et al. [62], and modelled the occurrence probability for species *i* at sampling point *l* by incorporating point-specific habitat characteristics. We incorporated willow area, edge density, willow height and willow density in the occupancy estimates by assuming that the logit transform of the occurrence probability (*ψ*) was a linear combination of a species effect (*i*) and the point-specific (*l*) willow configuration in each year (*j*) as follows:
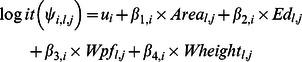



The willow configuration variables were standardized (mean = 0, SD = 1), meaning that the inverse-logit of *u_i_* is the occurrence probability for species *i* in sampling points with mean values of the willow configuration variables. Moreover, the *β_i_*’s are the effects of the different willow configuration variables for species *i*. The detection probability (*p*) for species *i* was assumed to vary based on the same willow configuration variables:
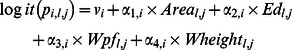
where *v_i_* refers to the detection probability of species *i* for mean values of the willow configuration variables. Our analysis was performed using WinBUGS 1.4.3 [65], which uses Markov Chain Monte Carlo (MCMC) simulations to estimate posterior probability distributions. We estimated the model parameters as well as community summaries (i.e. total, yearly and assemblage-specific species richness) by using the same uninformative prior distributions for all parameters as those used by Kéry et al. [62]. Note that the community summaries are calculated as the sum of species specific occupancy rates for each sampling point (i.e. the specific values of the willow configuration variables) each year. Hence, as the estimates of species richness are derived from the occupancy models, we did not perform formal statistical analyses of the relation between species richness and willow configuration variables (cf. [66]). To allow for potentially present, but non-detected, species when estimating measures of community (or assemblage) richness we followed the data augmentation procedure presented by Royle and Dorazio [67]. Thus, we added M-n = 32 (n = 17 species detected) all-zero species and ran two parallel chains of length 30000 from random starting values, discarded the first 3000 as burn-in, and retained 1 in 10 updates. Model convergence was assessed by the convergence factor Rhat for each parameter in the model (i.e. Rhat close to 1 implies convergence [68]).

Finally, we estimated relative species abundance. Abundance is a metric that may be more relevant than mere probability of presence (i.e. occupancy) in considerations of the function of species in ecological communities, and is moreover methodologically important since abundant species are likely to be easier to detect across the landscape. We used the single-species hierarchical model of Royle [69], i.e. the N-mixture model, to estimate relative abundance of the individual species based on the spatially replicated bird counts. In the N-mixture model, point-specific abundance is treated as a random effect, where the marginal likelihood of the counts is obtained by integrating the binomial likelihood for the observed counts over possible values of abundance for each point [69]. Note that estimated abundance from the N-mixture model reflects the average number of individuals per sampling point. We included the willow configuration variables as covariates for abundance and study years as covariate for detection (see Table S1 in Appendix S1). The analysis of species-specific abundance was performed using the function *pcount* in the package *unmarked* [70] in R [71]. Finally, we calculated assemblage-specific estimates of the covariates of abundance using the function *metagen* (i.e. the fixed effects model) in package *meta* in R [71]. The function *metagen* can generally be applied to all types of data as long as estimates of the effect size and corresponding estimated standard errors are given.

## Results

### Bird Community

Throughout our 4 year study 17 species were detected at least once in our surveys (Table 1). Nine species were *a priori* classified as willow related species (4 Willow Canopy-Breeding (WCB) and 5 Willow related Ground-Breeding (WGB) species), whereas 8 species were not expected to be related to willow thickets (i.e. Open Tundra (OT) representing the heath and meadow vegetation states). Willow-dependent species (both WCB and WGB) were predominantly observed in the willow thickets, while most of the typical willow independent species (OT) were predominantly observed in the surrounding heaths and meadows (Table 1). Exceptions were the Red-throated Pipit, Meadow Pipit, White Wagtail and Lapland Bunting, which were also frequently observed in relation to the willow thickets. Besides two open tundra species, the Meadow Pipit and Lapland Bunting, the most numerous species were those affiliated with willow thickets (Table 1).

### Species Richness

**Figure 1:**
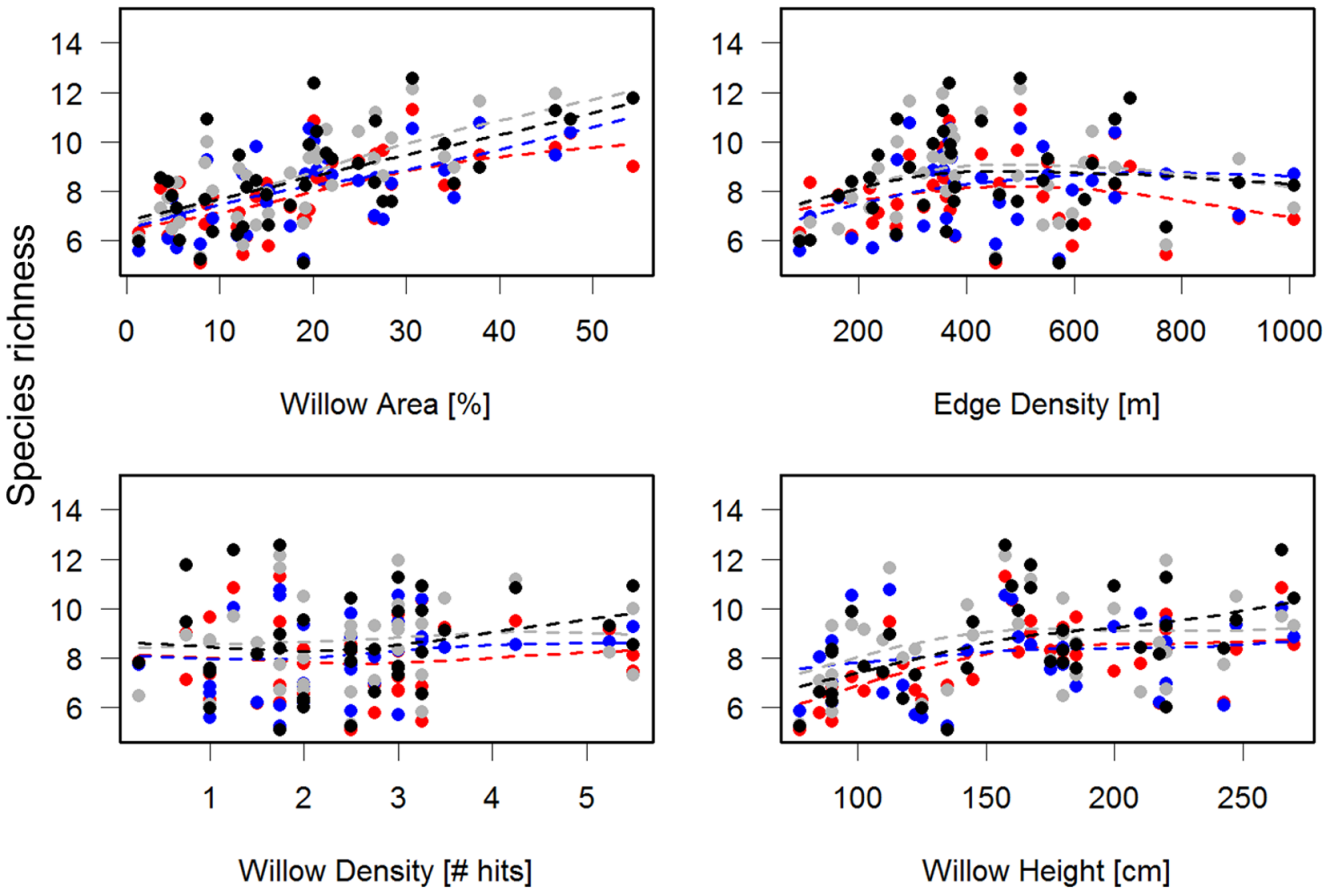
Relationships (i.e. marginal effects) between total yearly point-specific species richness (y-axis) and willow configuration variables. Red points and lines denote 2005, blue denote 2006, grey denote 2007 and black denote 2008. The solid lines correspond to a smoothing spline with 3 df. Note that the smoothing splines are only included to ease interpretation with respect to the direction of the relationships.

**Figure 2:**
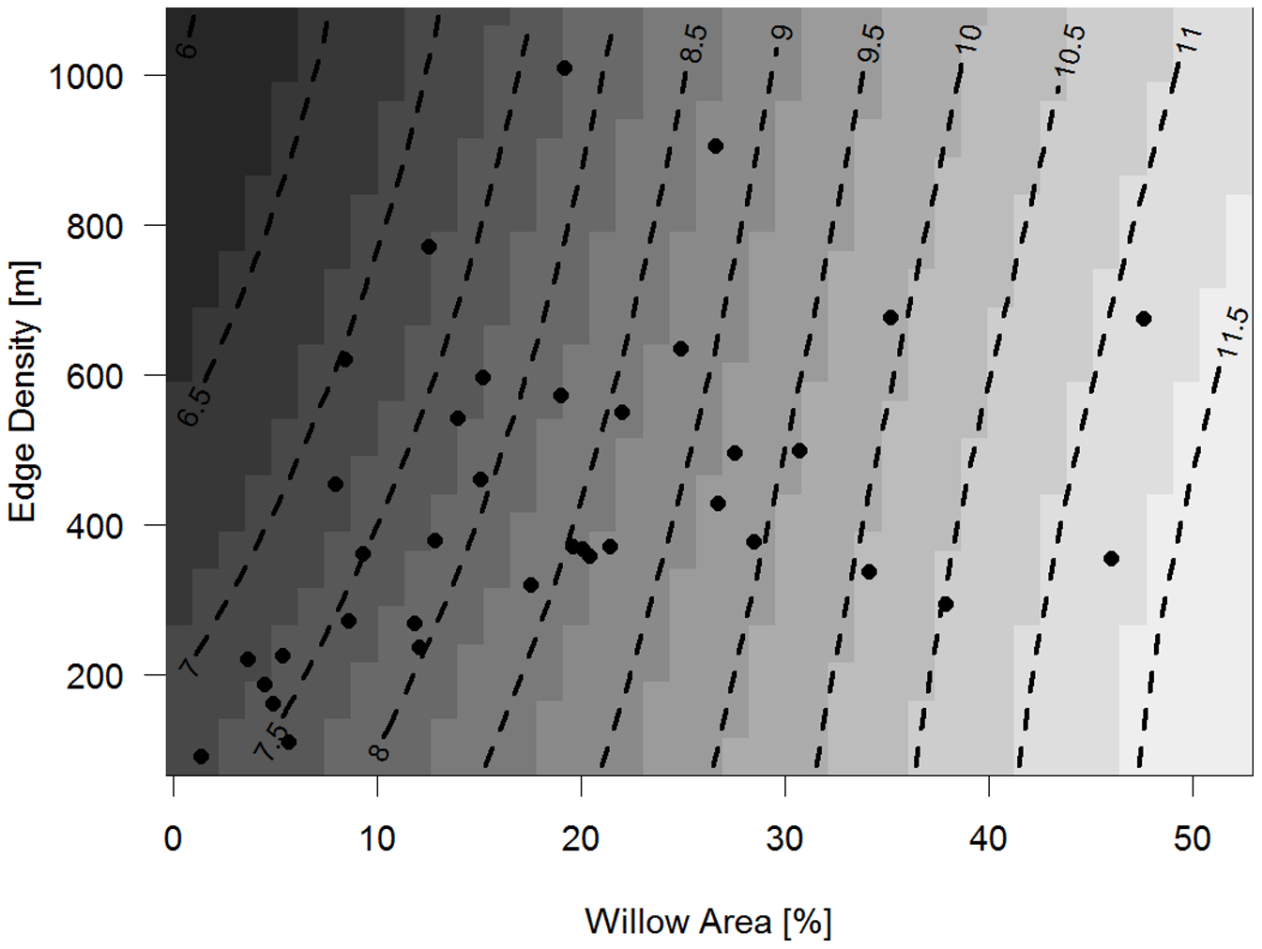
Estimated species richness at the sampling points as a function of willow area and fragmentation (i.e. edge density). Stippled lines with numbers and shading denote estimated species richness, whereas the points denote the measured values of willow area and edge density for all sampling points. Note that the figure depicts the conditional effect of willow area and fragmentation.

The total species richness over the four year study was estimated to be 18.3 (95% CI: [17], [24]) species, with a range in total richness (i.e. sum over the 4 years) at the sampling points between ∼10 and 16 species (Figure S1 in Appendix S1). The estimated yearly point richness showed some clear and temporally consistent patterns with respect to willow area and configuration (Figure 1). The most profound effect (i.e. marginal) was a strong linear increase in the estimated point richness with increasing area of willows. This response corresponds to a decrease of ∼ 4 species out of 12 when willow area extent reduces from 50% to ∼ 5% (Figure 1). A weak positive relationship was also present for willow height. For edge density there was an apparent weak concave relationship with species richness. However, this apparent pattern was influenced by the fact that both low and high degree of fragmentation corresponded to low willow area (i.e. conditional effect: Figure 2). Hence, the effect of fragmentation was somewhat confounded with the effect of willow area, even though species richness was maximized in patches of intermediate degree of fragmentation (Figure 2). In sum, yearly species richness was highest in areas with high willow cover of small-intermediate degree of fragmentation (amounts of edge habitat) (Figure 2). Finally, there was no relationship between willow density and yearly point richness (Figure 1).

By decomposing the yearly species richness into richness within the species assemblages (Figure S2, S3 and S4 in Appendix S1,), we found that the positive effect of area was mostly driven by an increase in willow-dependent species, especially WGB. The weak positive effect of willow height on yearly richness was mainly due to changes in the community of willow related ground-breeding species (WGB). Finally, the assemblage of species with a looser connection to willow thickets (OT) showed no consistent responses in richness to willow area, fragmentation or local habitat variables.

### Species Occupancy, Detection and Abundance

**Figure 3:**
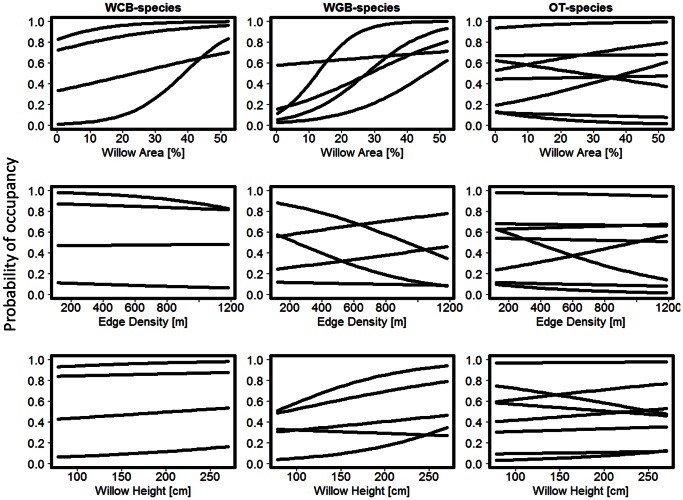
Probability of occupancy as a function of willow area and configuration variables for the three different species assemblages. WCB denote Willow Canopy-Breeding, WGB denote Willow related Ground-breeding and OT denote Open Tundra species. The relationships between species occupancy and willow density are not depicted. Note that the different curves represent individual species responses within each assemblage.

The estimated mean probability of occupancy varied widely among species, ranging from ∼ 7 to 95% (see Appendix S2). Mean detection probability was quite low for many species (e.g. 7 species with <20% detection probability) and varied a lot among species (∼12–71%, Appendix S2). Moreover, there was a high positive correlation between estimated occupancy and detection (r = 0.75, see Figure S5 in Appendix S1). With respect to the effect of willow area and configuration, willow area had a large impact on estimated occupancy for many species within the community (Figure 3). Of the 17 species observed, 10 displayed distinct positive relationships between the probability of occupancy and willow area, whereas only one species showed a distinct negative relationship (i.e. Golden plover). With respect to the species assemblages, willow-dependent species (WCB and WGB) displayed a significant positive response to increasing willow area, whereas the willow independent species did not (Table 2). Fragmentation (i.e. edge density) had no statistically significant impact on estimated occupancy probability, and showed much variation both among and within the species assemblages (Figure 3 and Table 2). Only one species displayed a significant positive relationship between occupancy and willow height (Figure 3) and consequently there was no significant mean response for any of the assemblages (Table 2). No species displayed strong relationships to change in willow density and there were no significant mean response for any of the assemblages (Table 2).

**Table 2:** Estimates of the average occupancy (logit-scale) and abundance (log-scale) response to willow configuration variables for the willow-dependent (WCB & WGB) and willow independent species (OT) assemblages.

	Assemblage WCB & WGB			Assemblage OT		
Occupancy	median	95% Credible Intervals		median	95% Credible Intervals	
Area	**0.99**	**0.55**	**1.57**	0.01	−1.34	1.61
Edge density	−0.13	−1.15	0.74	−0.09	−1.07	0.75
Willow density	0.09	−0.72	0.62	0.10	−0.58	0.69
Willow height	0.30	−0.52	1.27	0.09	−0.87	0.99
**Abundance**	**mean**	**95% CI**		**mean**	**95% CI**	
Area	**0.27**	**0.20**	**0.34**	−0.10	−0.21	0.01
Edge density	0.02	−0.06	0.10	−0.01	−0.13	0.10
Willow density	0.01	−0.06	0.08	0.08	−0.02	0.17
Willow height	**0.16**	**0.08**	**0.23**	−0.04	−0.15	0.07

The upper part depicts average assemblage estimates for the predictors of occupancy, while the lower part show average estimates for the predictors of abundance. The estimates of average assemblage occupancy and confidence intervals are given as the median and 95% credible intervals, respectively. The estimates of assemblage-specific abundance were estimated using the function metagen in R [71]. Note that accounting for heterogeneity (i.e. random effect model) in the estimation of abundance did not change the results, only resulting in slightly lowered mean and wider confidence intervals. Numbers in bold indicate significant effects.

Estimated relative abundance varied greatly (range on log-scale: −3.75 to 3.86) within the bird community (see Table S1 in Appendix S1). This high heterogeneity in abundance may thus be part of the explanation for the strong correlation between occupancy and detection [cf. 45,72]. Indeed, species with high abundance generally showed higher estimated detection (r = 0.51) and occupancy probabilities (r = 0.52) (see Table S1 in Appendix S1, Appendix S2). Except for the Meadow Pipit, species belonging to WCB and WGB were overall the most abundant species (see Table S2 in Appendix S1), which was, except for the Lapland Bunting, in line with the raw count data (Table 1). The assemblages of willow-dependent species displayed significant positive abundance relationships with willow area and willow height, but not to edge or willow density (Table 2). In contrast, the assemblage of willow-independent species did not display significant abundance responses to any of the willow variables (Table 2).

## Discussion

### Synthesis of Results

Quantifying the effects of spatial variation in mosaics of riparian vegetation states on bird communities in low arctic tundra, we found that species occupancy and richness were greatly affected by willow thicket areal extent, configuration and local habitat structure, i.e. habitat features likely to be affected by intense browsing by ungulates as well as climate warming. These estimated effects appear to be robust as they were consistent over the 4-year study period and across three separate riparian regions. Especially, species richness showed a strong positive relationship with willow thicket area and to a lesser extent the height of willow thickets. Willow fragmentation had an almost negligible effect on species richness and was overshadowed by the strong effect of area. In sum, we found that total species richness was maximized in large (and tall) willow patches of intermediate degree of fragmentation. These community effects were mainly driven by changes in the assemblages we *a priori* defined as willow-dependent species, especially the willow ground-breeding species (WGB). The assemblage of species that according to prior knowledge was not expected to be connected to thickets displayed no responses in richness to changes in willow area and configuration, although some of these species were frequently observed in the thickets. However, Ims and Henden [11] found that even open tundra (OT) species may be severely affected by total loss of tall willows in the riparian habitats owing to high abundance of browsing ungulates. Thus even a few small patches of tall willow, only making up as little as 5% of the riparian habitat (i.e. near the minimum sampled in the present study), provide a positive significant function for bird species. Both occupancy and detection varied substantially within the bird community. With respect to willow configuration, willow area, height and fragmentation had a large impact on species-specific estimates of occupancy for many species within the community. However, at the level of the species assemblages, only willow area showed a significant relationship to occupancy, and only for *a priori* defined willow-dependent species.

### Effects of Habitat Configuration on Community Richness and Species Occupancy

The observed number of species (i.e. 17) in this study conforms well to that found in a survey conducted 30 years ago in the same region of north-eastern Norway (21 species; [73]). The estimate under our model of 18.3 (95% CI: [17], [24]) therefore appears sensible. The relationships between species richness and the predictors of willow area, configuration and structure were partly as expected; there was a strong decline as willow areas became smaller and lower in height. These results were brought about by a coherent positive response in the estimated occupancy probability of most species to areal extent and to some extent willow height. The effect of fragmentation was strongly confounded with the area effect, in the sense that both high and low degree of fragmentation coincided with low habitat area. Thus, the marginal effect of fragmentation was close to negligible. This is in accordance with Mortelliti et al. [74] who found that the amount of forest cover in the landscape had the strongest relative influence on birds’ occupancy, whilst habitat subdivision played a negligible role. Yet, the diversity of species responses in occupancy to increased fragmentation likely promoted the slight increase in species richness seen at intermediate levels of fragmentation seen in figure 2. This lends some support to the patch heterogeneity hypothesis (c.f. [49]), in which richness or abundance is expected to be highest at intermediate levels of fragmentation. On the other hand, the relative effect of area and fragmentation is obviously difficult to separate in natural landscapes, since willow thickets in our large scale study are confined not only by ungulate browsing, but also local topography (i.e. valleys). This clearly contributes to the observed pattern seen in figure 2, where none of the sites harboured a combination of high areal extent and very high or low degree of fragmentation of willow patches, thus likely enforcing such patterns of high richness in patches of intermediate fragmentation in natural landscapes. To be fully able to tease apart fragmentation and area effects one would need manipulative experiments at spatial scales that would be logistically and ethically unacceptable in the focal ecosystem.

Evidently willow shrubs provide important functions to many of the bird species in low arctic tundra. The positive effect of increased areal extent of thickets could thus be expected based on the importance of willows as structural elements in the otherwise barren tundra both as refuges from predators and breeding and foraging habitat [11], [75]. Indeed, Ims & Henden [11] found that more than half of the bird species were lost where willow thickets had been entirely removed by intense ungulate browsing. In that case even some open ground habitat specialists appeared to be negatively impacted, likely due to halted spatial spillover of food resources from the rich fauna of insects dwelling in the willow thickets [76]. Thus, negative effects on open tundra specialists may not be expected before willow thickets disappear entirely from riparian habitats, possibly explaining the lack of response of OT species in the current study. Several previous studies have shown that the effect of habitat area in fragmented landscapes is consistently positive and strong across regions, habitats and taxa [41], [43]–[45], [47]. Hence, large thicket complexes likely harbour more resources both for breeding (e.g. breeding locations) and foraging (e.g. insects) and thereby an increased capacity to support more species and breeding pairs [77]. This will clearly increase the detection of at least one territory of a species with greater likelihood in large compared to smaller patches (c.f. [78]). Indeed, the probability of detection increased with areal extent of willow thickets for many species (i.e. 8 of 17 species).

Decomposing the estimates of total species richness into the *a priori* species assemblages, we found that most of the relationships were driven by changes in the two groups of willow-dependent species, especially the willow ground-breeding species. In terms of mechanisms, the effect of areal extent of willow thickets on especially willow ground-breeding birds in our study is likely related to predation, the primary agent of avian nest mortality [79], [80]. In our study areas, several predators of small to medium sized birds, both avian (e.g. raven *Corvus corax*, Hooded crow *Corvus cornix* and Long-tailed skua *Stercorarius longicaudus*) and mammalian (e.g. red fox *Vulpes vulpes*, stoat *Mustela erminea* and least weasel *Mustela nivalis*), are present during the breeding season. Several studies have reported elevated rates of predation in fragmented landscapes, small habitat remnants and along habitat edges [79], [80]. Accordingly, some studies have found that avian [79]–[82] as well as some mammalian (e.g. stoat and foxes; [83] and references therein, [84]) predators are more common along habitat edges than in the habitat interior. Thus, a high predation pressure might promote an increased aggregation of birds in larger and more homogenous patches, as large homogeneous patches of willows are likely to reduce the accessibility to patch interiors of especially avian, but also mammalian, predators.

Under the predicted climate driven shrub encroachment of tundra [22], [85] our study shows that most bird species related to riparian willow habitats could be expected to benefit. While the willow shrub state may rapidly replace the meadow state of the riparian plains in a warmer climate, the meadows appeared to be little used by the focal bird community compared to willow thickets and heaths (Table 1). Short-statured vegetation will still remain available for open tundra specialists in the oligotrophic heath habitats, at least until trees finally encroach on this stratum of the tundra landscape. The latter processes is however, expected to be much slower than the encroachment of shrubs in eutrophic riparian habitats (Sturm 2010). On the other hand, in parts of the Eurasian Arctic tundra, where ungulates, such as reindeer, have increased substantially during the last 20–30 years [15], [26], [34], [35], there is evidence that heavy grazing may be the dominating impact on willow thicket areal extent. For instance, from Fennoscandia there is evidence that heavy grazing by reindeer may significantly control deciduous shrub growth [32], [36], and currently prevent the disappearance of short-statured tundra vegetation [37]. Hence, our study provides further support to the hypothesis that large herbivores may impact species and diversity negatively [6], [25], [75]. Our results clearly show that if willow thickets become extensively reduced in terms of areal extent many species will decline and possibly disappear.

## Supporting Information

Appendix S1 Supporting tables and figures.
(DOCX)

Appendix S2 Species specific estimates of occupancy, detection, and slope estimates of area and fragmentation variables.
(XLSX)
