# Supplementary material for: How Spatial Variation in Areal Extent and Configuration of Labile Vegetation States Affect the Riparian Bird Community in Arctic Tundra
Source: PLoS One. 2013 May 14;8(5):e63312. doi: 10.1371/journal.pone.0063312 (PMC3653927; doi:10.1371/journal.pone.0063312)
Supplement: Appendix S1 — Supporting tables and figures. (DOCX) [file pone.0063312.s001.docx]

Appendix S1

Table S1. Estimated relative abundance (i.e. average number of individuals per sampling point) of each species from the spatially replicated count data. For abundance the four willow configuration variables were included as covariates and for detection year was included. Significant (at the 95% level) estimates are highlighted in bold. Note that abundance is estimated as an “average” over the four years of the study.

Table S2. Mean and range of selected thicket configuration variables at the scale of 200x200m.

|  |  | **All regions** | | **Ifjord** | | **Komag** | | **V. Jakobselv** | |
| --- | --- | --- | --- | --- | --- | --- | --- | --- | --- |
| Variables | Measure | Mean | Range | Mean | Range | Mean | Range | Mean | Range |
| **Area** | % | 19.80 | [1.37,54.34] | 23.14 | [7.96,47.60] | 18.72 | [3.65,54.34] | 17.73 | [1.37,37.89] |
| **Edge density** | m | 440.13 | [91.66,1008.93] | 562.46 | [270.05,1008.93] | 391.28 | [188.46,703.62] | 372.29 | [91.66,676.51] |
| **Willow Height** | cm | 160.68 | [77.50,270.00] | 117.30 | [77.50,220.00] | 210.63 | [145.00,270.00] | 154.62 | [110.00,220.00] |
| **Willow Density** | no. of hits | 2.50 | [0.25,5.50] | 2.71 | [2.00,3.25] | 2.58 | [0.75,5.50] | 2.04 | [0.25,4.25] |


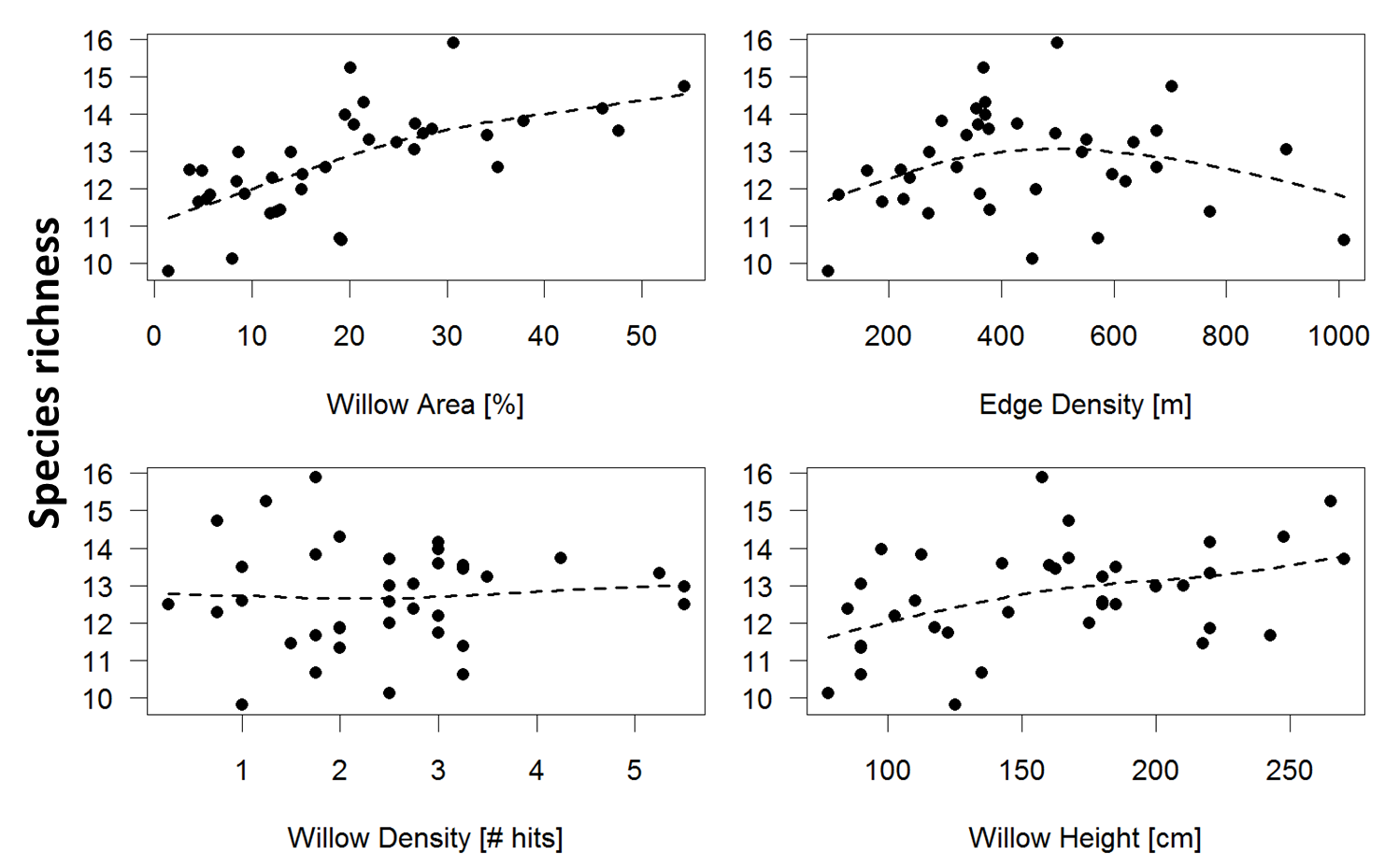


**Figure S1.** Relationships between total species richness at the sampling points and willow area and configuration variables. Upper left panel show the relationship between total richness and willow area, upper right show edge density in relation to total richness, lower left show willow density in relation to total richness and lower right show willow height in relation to total richness. Note that the smoothing splines are only included to ease interpretation of the direction in the relationships.


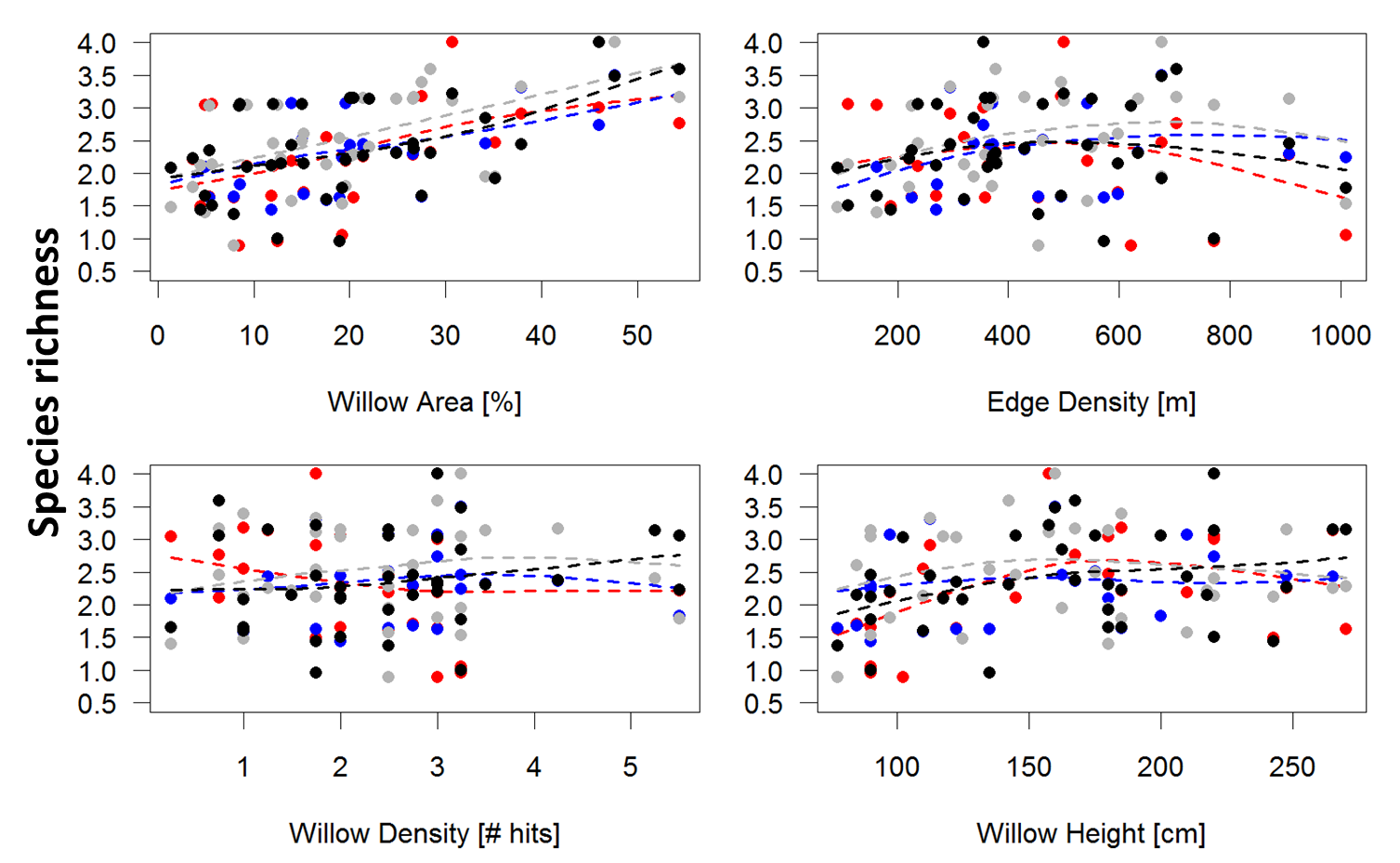


Figure S2. Relationships between yearly species richness and willow area and configuration variables for WCB. Upper left panel show the relationship between seasonal richness and willow area, upper right show edge density in relation to seasonal richness, lower left show willow density in relation to seasonal richness and lower right show willow height in relation to seasonal richness. Red points and lines denote 2005, blue denote 2006, grey denote 2007 and black denote 2008. Note that the solid lines correspond to a smoothing spline with 3 df. Note that the smoothing splines are only included to ease interpretation of the direction in the relationships.


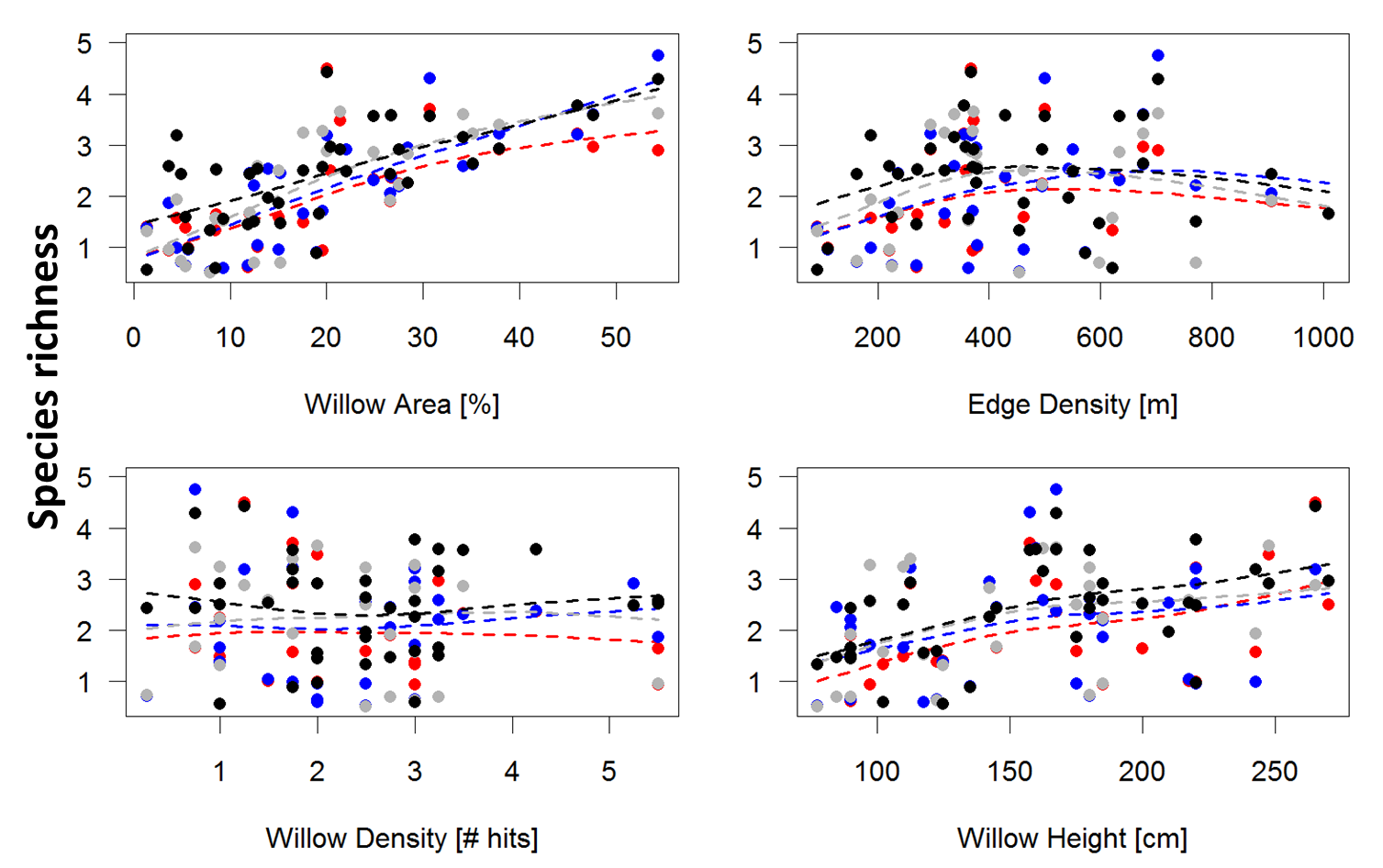


Figure S3. Relationships between yearly species richness and willow area and configuration variables for WGB. Upper left panel show the relationship between seasonal richness and willow area, upper right show edge density in relation to seasonal richness, lower left show willow density in relation to seasonal richness and lower right show willow height in relation to seasonal richness. Red points and lines denote 2005, blue denote 2006, grey denote 2007 and black denote 2008. Note that the solid lines correspond to a smoothing spline with 3 df. Note that the smoothing splines are only included to ease interpretation of the direction in the relationships.


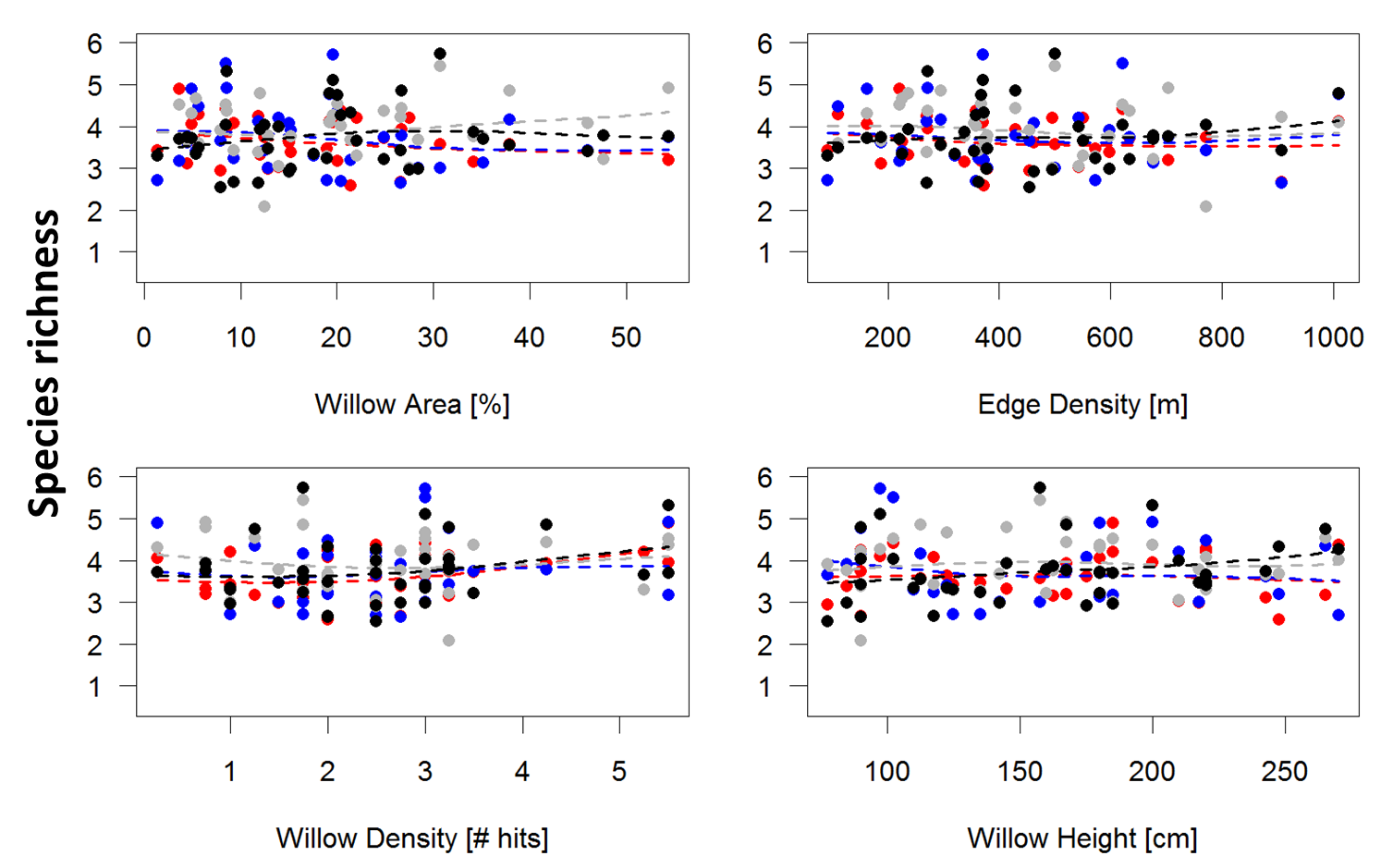


**Figure S4.** Relationships between yearly species richness and willow area and configuration variables for OT. Upper left panel show the relationship between seasonal richness and willow area, upper right show edge density in relation to seasonal richness, lower left show willow density in relation to seasonal richness and lower right show willow height in relation to seasonal richness. Red points and lines denote 2005, blue denote 2006, grey denote 2007 and black denote 2008. Note that the solid lines correspond to a smoothing spline with 3 df. Note that the smoothing splines are only included to ease interpretation of the direction in the relationships.

**Figure S5.** Mean estimated values of occupancy and detection for the 17 species that were observed during the course of the study. The stippled line shows the one to one relationship of equal occupancy and detection.
